# Neutral lineage tracing of proliferative embryonic and adult mammary stem/progenitor cells

**DOI:** 10.1242/dev.164079

**Published:** 2018-07-25

**Authors:** Bethan Lloyd-Lewis, Felicity M. Davis, Olivia B. Harris, Jessica R. Hitchcock, Christine J. Watson

**Affiliations:** 1 Department of Pathology, University of Cambridge, Cambridge CB2 1QP, UK; 2 Wellcome Trust-Medical Research Council Cambridge Stem Cell Institute, University of Cambridge, Cambridge CB2 1QR, UK

**Keywords:** Mammary gland development, Embryonic mammary stem cells, Adult mammary stem cells, Lineage tracing

## Abstract

Mammary gland development occurs over multiple phases, beginning in the mammalian embryo and continuing throughout reproductive life. The remarkable morphogenetic capacity of the mammary gland at each stage of development is attributed to the activities of distinct populations of mammary stem cells (MaSCs) and progenitor cells. However, the relationship between embryonic and adult MaSCs, and their fate during different waves of mammary gland morphogenesis, remains unclear. By employing a neutral, low-density genetic labelling strategy, we characterised the contribution of proliferative stem/progenitor cells to embryonic, pubertal and reproductive mammary gland development. Our findings further support a model of lineage restriction of MaSCs in the postnatal mammary gland, and highlight extensive redundancy and heterogeneity within the adult stem/progenitor cell pool. Furthermore, our data suggest extensive multiplicity in their foetal precursors that give rise to the primordial mammary epithelium before birth. In addition, using a single-cell labelling approach, we revealed the extraordinary capacity of a single embryonic MaSC to contribute to postnatal ductal development. Together, these findings provide tantalising new insights into the disparate and stage-specific contribution of distinct stem/progenitor cells to mammary gland development.

## Abstract (precis)

**Summary:** Neutral, low-density lineage tracing of proliferative mammary stem and progenitor cells during embryonic, pubertal and reproductive mammary gland development reveal the disparate and stage-specific contribution of distinct stem/progenitor cells.

## INTRODUCTION

Mammary gland development is a complex and multi-stage process that begins in the embryo and continues throughout the reproductive life of female mammals (Cowin and Wysolmerski, 2010; Gjorevski and Nelson, 2011; Hinck and Silberstein, 2005). This process commences with the formation of two milk lines from the overlying ectoderm on embryonic day (E) 10.5 in mice, and the asynchronous appearance of five pairs of placodes at specific and symmetric locations between the fore- and hindlimbs by E11.5 (Hens and Wysolmerski, 2005). These ectodermal placodes develop asynchronously and invaginate to form the mammary bud by E13.5, followed by the formation of an epithelial tubular sprout by E15.5-16.5 that invades the underlying mammary fat pad precursor. Contact with the developing fat pad initiates a phase of branching morphogenesis, resulting in the formation of the primordial ductal tree by E18.5, prior to birth (Veltmaat, 2017; Veltmaat et al., 2003; Watson and Khaled, 2008).

In the weeks immediately after birth, growth of the ductal tree is commensurate with body growth and it is not until puberty that ductal structures begin to elongate rapidly and invade the empty fat pad, driven by hormonal and growth factor signalling in the micro-environment (Hinck and Silberstein, 2005). This process, known as ductal morphogenesis, is orchestrated by proliferation of adult mammary stem and progenitor cells within the distal terminal end bud (TEB) structures (Bai and Rohrschneider, 2010; Paine et al., 2016; Sreekumar et al., 2015). After pubertal growth is complete, the mammary epithelium re-enters a phase of balanced proliferation, with only minor growth and remodelling occurring with cyclical ovarian hormone stimulation. However, rapid expansion of the epithelium again occurs during pregnancy and lactation, when adult MaSCs proliferate to form lobuloalveolar structures capable of producing and expelling milk for neonatal nourishment (Davis et al., 2016; Lloyd-Lewis et al., 2017; Sreekumar et al., 2015).

Despite their essential role in pre- and postnatal mammary gland development, studies to determine the molecular identity and differentiation potential of MaSCs have yielded conflicting results (for recent reviews, see Sreekumar et al., 2015; Lloyd-Lewis et al., 2017). Recently, both saturation and single-cell genetic lineage-tracing studies have demonstrated that lineage-restricted MaSCs appear to drive postnatal mammary development under physiological conditions (Davis et al., 2016; Scheele et al., 2017; Wuidart et al., 2016). However, these studies also demonstrated significant redundancy and heterogeneity within the adult MaSC compartment, and the differential and stage-specific contribution of diverse stem/progenitor cells in the breast is still emerging (Bach et al., 2017; Cai et al., 2017; Van Keymeulen et al., 2017; Wang et al., 2017). In this study, we employed a low-density, neutral, genetic labelling strategy to further investigate the extent and nature of the contribution of proliferative stem/progenitor cells to embryonic, pubertal and reproductive mammary development.

## RESULTS AND DISCUSSION

### A pool of lineage-biased adult stem/progenitor cells propel ductal elongation during puberty

Recently, genetic lineage-tracing studies in the mouse mammary gland have achieved *in vivo* indelible marking of specific populations of cells (characterised by their expression of nominated genes at specific developmental stages) and the subsequent analysis of the progeny of proliferative labelled cells after an appropriate chase (Sale and Pavelic, 2015). Targeted cell populations include those temporally or stably expressing: keratin (K) 5 (Rios et al., 2014; Van Keymeulen et al., 2011), K14 (Rios et al., 2014; Tao et al., 2014; Van Keymeulen et al., 2011; Wuidart et al., 2016), K8 (Tao et al., 2014; Van Keymeulen et al., 2011; Wuidart et al., 2016), K18 (Van Keymeulen et al., 2011), K19 (Wuidart et al., 2016), Elf5 (Rios et al., 2014), Lgr5 (de Visser et al., 2012; Fu et al., 2017; Rios et al., 2014; Van Keymeulen et al., 2011; Wuidart et al., 2016), Lgr6 (Blaas et al., 2016; Wuidart et al., 2016), Sox9 (Wang et al., 2017; Wuidart et al., 2016), Axin2 (van Amerongen et al., 2012), Notch1 (Rodilla et al., 2015), Notch2 (Šale et al., 2013), Notch3 (Lafkas et al., 2013), WAP (Chang et al., 2014), Acta2 (Prater et al., 2014), p63 (Sreekumar et al., 2017), Procr (Wang et al., 2015), prominin 1 (Wang et al., 2017) and ER (Van Keymeulen et al., 2017). However, although providing valuable information on mammary development and the epithelial differentiation hierarchy, these models have relied on prior assumptions regarding the specificity and consistency of the expression of the chosen gene promoters, and have generated conflicting results.

**Fig. 1.**
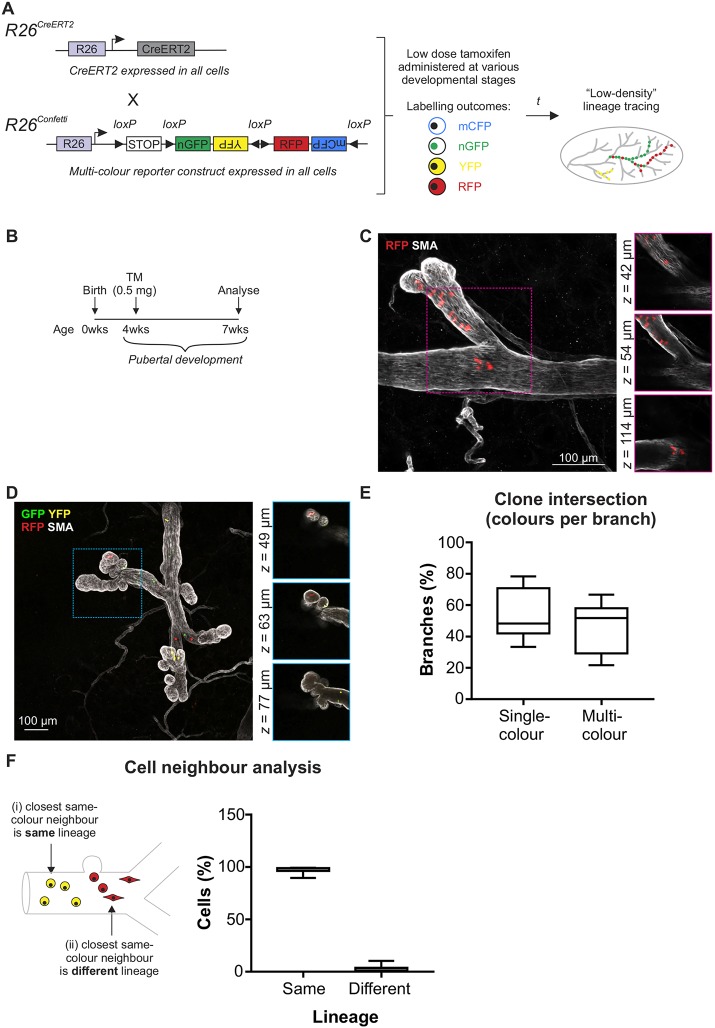
**Lineage tracing during branching morphogenesis.** (A) The *R26^CreERT2^;R26^Confetti^* model. *R26^CreERT2^* mice (expressing inducible Cre-recombinase in all cells) were crossed to *R26^Confetti^* mice (expressing a conditional multicolour reporter in all cells) to generate double hemizygous mice. Administration of low-dose tamoxifen produced stochastic genetic labelling of cells at relatively low density. Labelling outcomes include membranous CFP (mCFP), nuclear GFP (nGFP), cytosolic YFP (YFP) or cytosolic RFP (RFP); however, CFP^+^ clones (Fig. S2) were under-represented (Davis et al., 2016) and were not analysed. (B) For lineage tracing during branching morphogenesis, tamoxifen was administered (4 weeks) and tissue harvested (7 weeks). (C,D) Example of single-colour branches (C) and multicoloured branches (D). Images show maximum-intensity *z*-projections and optical slices of a region of interest (boxed and enlarged in the right-hand panels). (E) The percentage of single- and multicolour branches in pubertal mice. (F) Cell neighbour analysis revealed that the majority of FP^+^ cells had a same-colour FP^+^ neighbour that was the same lineage. Box extends from the 25th to 75th percentiles and whiskers indicate minimum to maximum values. Data are from 1419 cells distributed across 130 branches from randomly selected 3D images (five mice).

In this study, we have employed a neutral genetic labelling strategy for lineage analysis in the mammary gland using *R26^CreERT2^;R26^Confetti^* mice (Fig. 1A) (Davis et al., 2016; Li et al., 2016; Scheele et al., 2017). Administration of a low dose of tamoxifen induces the stochastic expression of up to four fluorescent proteins (FPs) (Fig. 1A). Importantly, FP expression can occur in any cell, overcoming issues pertaining to the requisite high-level Cre specificity inherent to other models (discussed by Wuidart et al., 2016; Davis et al., 2016 ; Lloyd-Lewis et al., 2017).

Neutral labelling of proliferative cells at clonal density (where the chance of clone convergence is extremely low) has previously been described using the *R26^CreERT2^;R26^Confetti^* model (using an ‘ultra-low’ dose of tamoxifen; 0.2 mg per 25 g body weight) (Scheele et al., 2017) and the *R26^[CA]30^* model (Davis et al., 2016). Using these models combined with 3D imaging, all of the progeny of a single labelled cell can be analysed with confidence. These studies revealed that lineage-restricted stem/progenitor cells orchestrate ductal (Davis et al., 2016; Scheele et al., 2017) and alveolar (Davis et al., 2016) mammary morphogenesis. However, they also revealed extraordinary multiplicity in the MaSC compartment and thus their power to capture the full spectrum of mammary stem/progenitor cells is limited.

In the current study, we injected pubertal *R26^CreERT2^;R26^Confetti^* mice with 0.5 mg tamoxifen (∼35 µg/g) to achieve low-density labelling in the mammary epithelium (Fig. 1B and Fig. S1A). This dose is approximately fourfold higher than previous studies using ‘ultra-low’ tamoxifen dosing in puberty (Scheele et al., 2017). Using this approach, we observed mammary branches that contained labelled cells of a single colour (Fig. 1C) as well as branches comprising two or more colours (Fig. 1D), as expected. No labelling was observed in control vehicle-injected mice (Fig. S1B). Quantification of the number of single- and multicoloured branches indicated that, under these conditions, the likelihood of clone convergence is at least 50% (Fig. 1E); this number may be even higher, as distinct coincident labelling events of the same colour cannot be distinguished.

Consistent with previous reports (Davis et al., 2016), we observed stochastic dispersion of labelled cell progeny throughout the developing ducts (Fig. 1C,D and Fig. S3). This labelling pattern is likely to have arisen from the deposition of labelled progeny along developing ducts by proliferative labelled cells in elongating TEBs (Bai and Rohrschneider, 2010; Davis et al., 2016). As ductal elongation and side branching occur as the result of cell proliferation by stem/progenitor cells within both TEB and ductal structures and the admixing of clonal progeny (Fu et al., 2017; Rios et al., 2014; Wang et al., 2015), we employed a cell-neighbour analysis to assess lineage potential (Fig. 1F; see supplementary Materials and Methods). A striking majority of same-colour cell neighbours consisted of cells of the same lineage, providing further evidence of physiological lineage bias in the postnatal mammary gland (Davis et al., 2016; Scheele et al., 2017; Van Keymeulen et al., 2011; Wang et al., 2017; Wuidart et al., 2016).

### Alveolar morphogenesis is driven by a pool of lineage-biased adult stem/progenitor cells

**Fig. 2.**
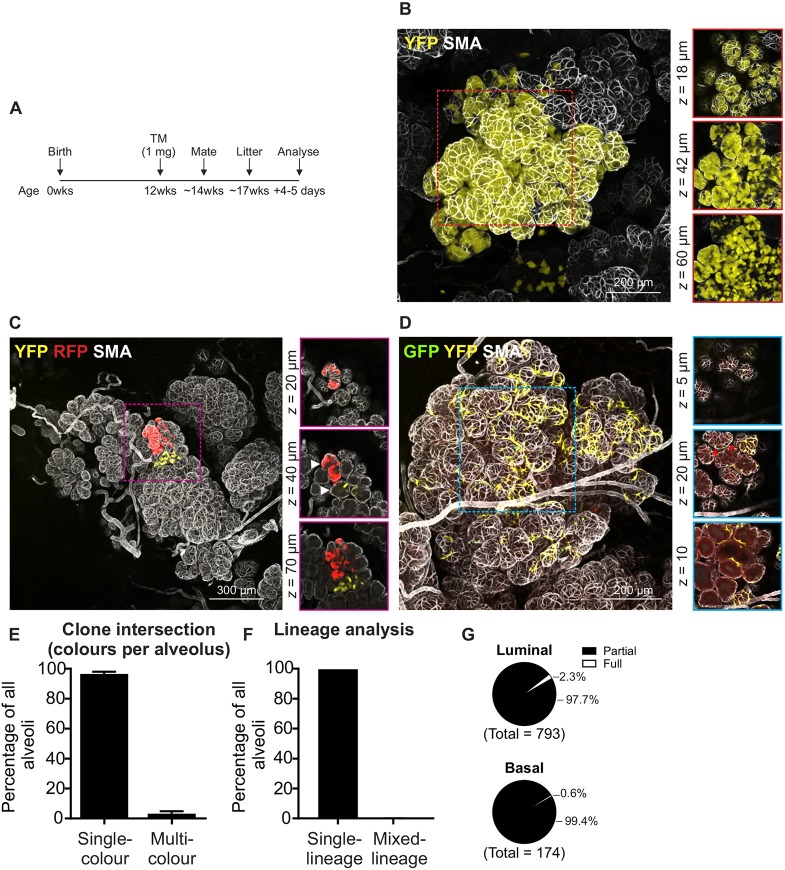
**Lineage tracing after alveolar morphogenesis.** (A) Tamoxifen was administered (∼12 weeks), mice were mated (∼14 weeks) and tissue harvested during lactation. (B-D) Example of single-colour luminal YFP^+^ alveoli (B), single-colour luminal YFP^+^ and RFP^+^ adjacent alveoli within a larger lobuloalveolar structure (arrowheads indicate different alveoli) (C) and multicolour basal GFP^+^ and YFP^+^ alveoli (D; red arrows show GFP^+^ and YFP^+^ cells within a single alveolus). Images show maximum-intensity *z*-projections and optical slices of a region of interest (boxed and enlarged in the right-hand panels). (E) Graph (data are mean±s.e.m.) showing the percentage of single- and multicoloured alveoli; a lower rate of clone convergence is observed in this model following expansion during gestation and lactation. (F) Graph (data are mean±s.e.m.) showing the percentage of FP^+^ alveoli in which the same-colour cells were the same lineage (i.e. all luminal or all basal) or where same-colour cells were mixed lineage (both luminal and basal). (G) Fraction of alveoli that were fully populated by single-colour FP^+^ cells of a single lineage (full) versus those populated by both single- or multicoloured FP^+^ cells and/or unlabelled cells of a single lineage (partial). Data represent 1016 alveoli from randomly selected 3D images (three mice).

Low-density labelling using the *R26^CreERT2^;R26^Confetti^* model was also used for lineage analysis during alveolar morphogenesis (Fig. 2, Figs S4 and S5). *R26^CreERT2^;R26^Confetti^* mice were injected with low-dose tamoxifen (1 mg per mouse; ∼40-50 µg/g) (Fig. S5A), mated and tissue harvested during lactation (Fig. 2A). Under these conditions, a large number of single-colour alveoli were observed (Fig. 2B,C and Fig. S4) with fewer multicoloured alveoli (Fig. 2D). No FP^+^ cells were observed in control mice (Fig. S5B). Analysis of individual alveolar units revealed the vast majority (96.6%) of alveoli were single-coloured (Fig. 2E). Of the single-coloured alveoli, only 0.1% contained both luminal and basal cells of the same colour (Fig. 2F). Thus, these data support previous lineage-tracing studies using a different neutral model at single cell density showing lineage restriction during alveolar morphogenesis (Davis et al., 2016). Previous single cell lineage-tracing studies, which quantified only very large labelled clones (containing hundreds of labelled cells, with each clone presumably arising from a single MaSC), demonstrated that most alveoli comprise the progeny of a pool of lineage-restricted cells (Davis et al., 2016). Analysis of the number of partially versus fully populated alveoli in this low-density model revealed a seemingly higher rate of polyclonality (Fig. 2G). This is likely due to the inclusion of small, medium and large clones in the current study, representing the wider spectrum of stem and progenitor cell divisions.

### Neutral lineage tracing supports the presence of lineage-biased embryonic stem/progenitor cells by late embryogenesis

**Fig. 3.**
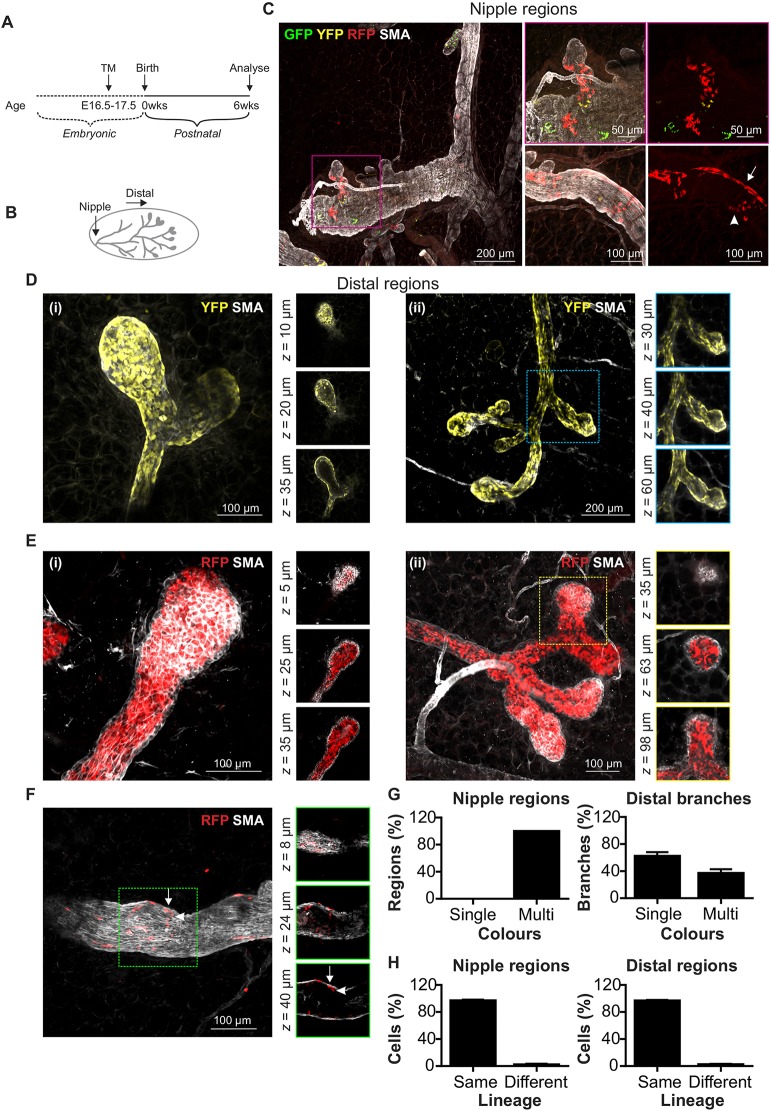
**Lineage tracing following embryonic labelling.** (A) Tamoxifen (33 µg/g maternal weight) was administered by oral gavage (E16.5-E17.5) and tissue harvested from *R26^CreERT2^;R26^Confetti^* offspring. (B) Analysis was divided into nipple regions and distal branches. (C) Example of a multicoloured nipple region. Arrow and arrowhead indicate adjacent RFP^+^ basal and luminal cells, respectively. (D) Example of single-colour distal branches and terminal end buds (TEBs) comprising YFP^+^ basal cells. i and ii are two examples showing the same thing. (E) Example of single-colour distal branches and TEBs comprising RFP^+^ luminal cells. (F) Example of a rare distal branch containing interspersed RFP^+^ luminal and basal cells. Images show maximum-intensity *z*-projections and optical slices of a region of interest (boxed and enlarged in the right-hand panels). Arrow and arrowhead in F show adjacent RFP^+^ basal and luminal cells, respectively. (G) Graphs (data are mean±s.e.m.) showing the percentage of single- and multicoloured nipple regions and distal branches. (H) Cell neighbour analysis showing that the majority of FP^+^ cells had a same-colour FP^+^ neighbour of the same lineage [data are mean±s.e.m. of 940 cells (seven nipple regions, *n*=5 mice) and 4439 cells (85 distal branches, *n*=7 mice) from randomly-selected 3D images].

Although MaSCs appear to be lineage restricted postnatally, numerous studies have suggested that their foetal precursors are multipotent (Boras-Granic et al., 2014; Fu et al., 2017; Rodilla et al., 2015; Spike et al., 2012; Trejo et al., 2017; Van Keymeulen et al., 2011; Wang et al., 2015). Embryonic MaSCs display multipotential activity in *in vitro* and transplantation assays, and increase dramatically in number during this developmental window (Spike et al., 2012). Moreover, *in vivo* population-based fate mapping has shown that all mammary epithelial lineages derive from embryonic K14-expressing stem/progenitor cells labelled at E17 (Van Keymeulen et al., 2011), an observation reinforced by other lineage-tracing studies using different gene promoters (Fu et al., 2017; Rodilla et al., 2015; Trejo et al., 2017; Wang et al., 2015). However, whether embryonic MaSCs are truly multipotent or whether they consist of populations of distinct progenitors that are already committed to give rise to different lineages after birth, has remained subject to deliberation. In addition, it has remained unclear when putative multipotent embryonic MaSCs become lineage restricted. Recent studies using low-density lineage tracing of embryonic cells have shed new light on this debate (Lilja et al., 2018; Wuidart et al., 2018). Clonal labelling of K14-expressing cells at E13 (when K14 appears to be universally expressed in luminal and basal lineages) points to the existence of multipotent stem cells at this stage of mammogenesis (Wuidart et al., 2018). However, at birth, segregation of basal and luminal lineages appears to be complete, with K5-expressing (Wuidart et al., 2018) and Acta2-expressing (Lilja et al., 2018) cells exclusively giving rise to basal progeny, and Notch1-expressing (Lilja et al., 2018) cells exclusively giving rise to luminal progeny. As targeted promoters may be differentially expressed in the neonatal and prenatal mammary gland (Sun et al., 2010; Boras-Granic et al., 2014; Trejo et al., 2017), definitive determination of the potential of embryonic MaSCs and their perinatal lineage segregation requires a neutral and inducible approach to labelling that is independent of these promoters. In addition, it has been demonstrated (Rios et al., 2016) that enzymatic digestion prior to 3D visualisation (Lilja et al., 2018; Wuidart et al., 2018) can alter tissue architecture and cell morphology, potentially confounding lineage-tracing outcomes. Unequivocal lineage determination must therefore include studies that employ methods of 3D visualisation that are void of proteolytic digestion (Lloyd-Lewis et al., 2016). To address this, a single low-dose of tamoxifen (33 µg per g maternal body weight) was administered by oral gavage to pregnant transgenic mice to induce incontrovertible neutral labelling in *R26^CreERT2^;R26^Confetti^* embryos at E16.5-E17.5. This route of delivery, although subject to first-pass metabolism, is reported to have less embryonic toxicity and more uniform recombination by Cre (Park et al., 2008). Mammary glands of offspring labelled *in utero* were subsequently visualised in 3D (without prior proteolytic digestion) (Fig. 3A). Using this approach, we observed large regions of labelled cells, some spanning from the nipple to the outer reaches of the fad pad (Fig. 3B-F, and Fig. S6). 3D imaging of areas proximal to the nipple revealed that these regions always comprised cells of two or more colours (Fig. 3C). Ductal branches in distal regions, however, more commonly contained cells of a single colour (Fig. 3D-F versus Fig. S7). Quantification of the number of single- and multicoloured branches in nipple and distal regions confirmed this observation, and indicated that the likelihood of clone convergence under these conditions was high (Fig. 3G). Mixed-lineage cells of the same colour were occasionally detected in both nipple (Fig. 3C) and distal (Fig. 3F) ductal regions, supporting the notion that embryonic MaSCs may possess multipotent capacity (Spike et al., 2012; Van Keymeulen et al., 2011). However, a cell neighbour analysis indicated that the majority of same-colour neighbours consisted of cells of the same lineage (Fig. 3H), suggesting that embryonic stem/progenitor cells are already lineage biased in the foetal mammary gland. Indeed, a recent study, which was able to achieve multicolour labelling at clonal density, has demonstrated that Notch1-expressing cells display lineage restriction at E15.5 and E17.5 (Lilja et al., 2018). It is important to note, however, that although the results of this study at E16.5-E17.5 are consistent with previous analyses at clonal density (Lilja et al., 2018; Wuidart et al., 2018), mammary glands in our study are marked at levels higher than clonal density and thus we cannot exclude the possibility that bipotent embryonic MaSCs were initially labelled and gave rise to luminal and basal progeny that expanded only after lineage specification occurred.

**Fig. 4.**
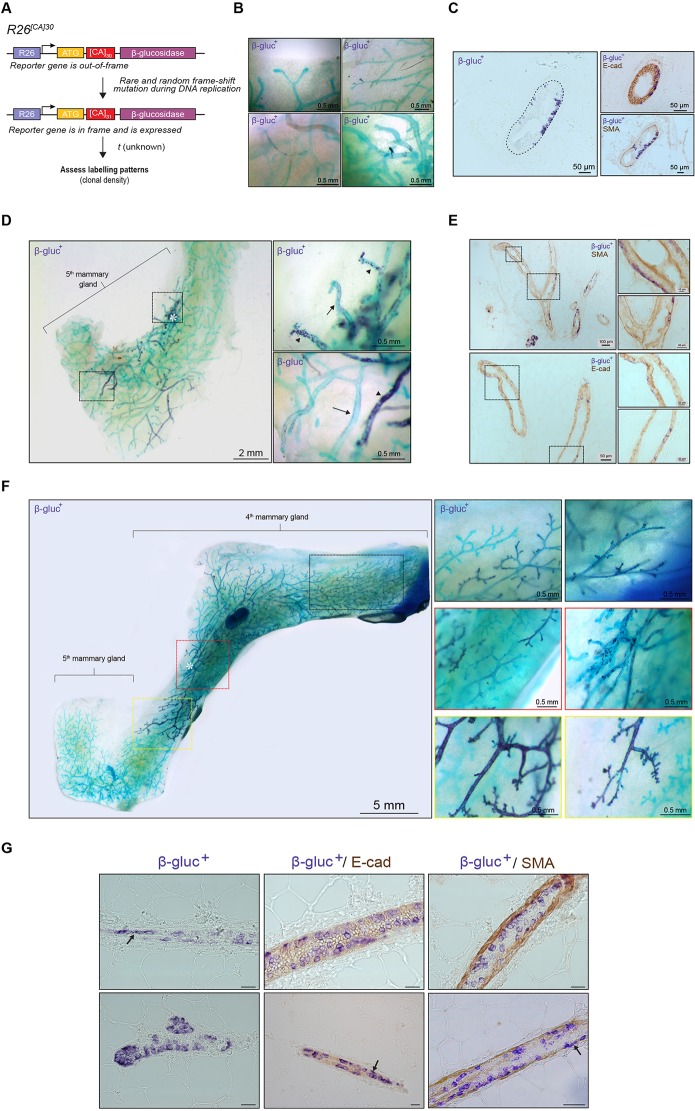
**Single-cell genetic labelling using the *R26^[CA]30SYNbglA^* model.** (A) Schematic representation of the *R26^[CA]30^* model. (B,C) Dispersion of β-glucosidase^+^ cells (purple) throughout the ductal epithelium (green) (B) and lineage analysis of β-glucosidase^+^ cells with immunohistochemistry (C). (D,E) Example of a β-glucosidase^+^ clone that originated at the nipple region (asterisk) (D) and consisted of regions containing β-glucosidase^+^ luminal cells adjacent to regions containing β-glucosidase^+^ basal cells (E). Arrows and arrowheads show cells with a basal and luminal morphology, respectively. (F) A rare clone spanning the entire abdominal mammary gland. Asterisk indicates the nipple region. (G) Lineage analysis of β-glucosidase^+^ cells with immunohistochemistry. Black arrows indicate β-glucosidase^+^ basal cells among β-glucosidase^+^ luminal cells. Scale bars: 20 µm. Clones shown in D and F represent two out of three putative embryonic labelling events observed from the analysis of 30 mice*.*

A single stem cell labelled *in utero* can contribute extensively to both the basal and luminal lineages in the adult mammary gland. Whereas the low-density and neutral *R26^CreERT2^;R26^Confetti^* model provided important corroborating evidence into the fate of primordial stem/progenitor cells to mammary development, we sought to reinforce these observations using an alternative neutral approach. To achieve this, we used the *R26^[CA]30^* reporter mouse model (Kozar et al., 2013) that was previously exploited to achieve unbiased, single-cell labelling in the mammary gland (Davis et al., 2016). This model encompasses a [CA]_30_ microsatellite repeat positioned directly upstream of an out-of-frame modified β-glucosidase (SYNbglA) reporter gene targeted to the Rosa26 locus (Fig. 4A). During DNA replication, spontaneous frame-shift mutations in the inherently unstable dinucleotide repeat tract may place the reporter gene in-frame, leading to its expression. This ‘strand slippage’ produces a permanent mark on the cell, which is subsequently transmitted to all of its progeny. Importantly, genetic labelling in this model is exceedingly rare, thereby allowing the fate of a single-labelled cell to be traced with a high degree of confidence.

Using this model, we observed variable numbers of label-positive cells randomly intermixed with unlabelled cells in developing ducts (Fig. 4B), mirroring the stochastic labelling pattern observed in pubertal *R26^CreERT2^;R26^Confetti^* mice (Fig. 1C,D). The majority of labelled progeny arising from a single β-glucosidase^+^ cell expressed markers of the luminal lineage (Fig. 4C), potentially reflecting the higher proliferative capacity in this compartment compared to basal cells (Giraddi et al., 2015). Isolated regions containing limited numbers of label-positive cells were most-commonly observed (Fig. 4B). These most likely arose from a recent frame-shift mutation, or from strand slippage in replicative-restricted progenitors or differentiated cells. Occasionally, large, contiguous clonal regions spanning several ductal branches were also observed, which were considered to have arisen from a single proliferative MaSC/progenitor cell.

On one occasion, we observed ductal regions that comprised exclusively β-glucosidase^+^ basal cells in close proximity to regions comprising only β-glucosidase^+^ luminal cells (Fig. 4D,E). The expansive size of this clone (>10 mm), in addition to its location at the nipple region of the mammary gland, suggest that a bipotent MaSC was labelled at some point during embryogenesis, giving rise to a luminal and a basal daughter cell that later generated lineage-restricted progeny during postnatal development. Remarkably, we also observed a clone that spanned the entire abdominal mammary gland (Fig. 4F). This is in stark contrast to the contribution of cells labelled during late embryogenesis in *R26^CreERT2^;R26^Confetti^* mice, which appeared, overall, more limited in scope (Fig. 3). The vast nature of this exceptional clone (and its origin at the nipple region) might, therefore, suggest that a cell was marked very early in development, likely before E12.5-E13.5, where clone size was considerably and consistently smaller (Lilja et al., 2018; van Amerongen et al., 2012; Wuidart et al., 2018). However, without knowing the precise stage of labelling in the *R26^[CA]30SYNbglA^* model, the differential contribution of these putative embryonic cells to ductal morphogenesis in this model (Fig. 4D versus 4F) could also point to heterogeneity and multiplicity within the embryonic MaSC compartment. Histochemical analysis of this rare clone (Fig. 4F) revealed that the majority of labelled cells were luminal (Fig. 4G), further suggesting that some MaSCs might exhibit a degree of lineage bias, even during early embryonic development.

The origin of luminal and basal cell lineages in the mammary gland has been the subject of intense investigation and debate. Recent saturation lineage-tracing, single-cell lineage-tracing and promoter-driven lineage-tracing studies have provided support for lineage restriction of MaSCs from late embryogenesis into adulthood (Davis et al., 2016; Li et al., 2016; Lilja et al., 2018; Van Keymeulen et al., 2011; Wuidart et al., 2016, 2018). However, the lack of evidence for the presence of multilineage clones does not unequivocally show that bi/multipotent stem cells do not exist (Rios et al., 2016; Visvader and Stingl, 2014). Thus, it is imperative that the epithelial hierarchy in the mammary gland is rigorously assessed at various developmental stages, using a range of methods, models and systems of analyses. Using a low-density, neutral, genetic labelling strategy and method of imaging that is free of proteolytic digestion, we have provided corroborating evidence of the lineage restriction of proliferative stem/progenitor cells to the three major stages of mammary development: in the late embryo, during puberty and in reproduction. Our findings also confirm that remarkable heterogeneity exists within the adult mammary stem and progenitor cell compartment, and suggest similar multiplicity within their embryonic precursors. Importantly, we have revealed the remarkable capacity of a single embryonic MaSC to contribute to ductal development, providing unprecedented insights that could only be disclosed by this single-cell approach. It is increasingly hypothesised that certain cancers may arise from reactivation of embryonic developmental programs in postnatal tissues (Howard and Veltmaat, 2013; Wahl and Spike, 2017). Thus, an elucidation of the full spectrum of stem/progenitor cell populations in the pre- and postnatal mammary gland is paramount for defining the cellular origin of heterogeneous breast tumours.

## MATERIALS AND METHODS

### Antibodies and reagents

Antibodies used in these studies include: rabbit anti-SMA (Abcam, ab5694, lot number GR248336-23, 1:200 and 1:300 for 2D and 3D studies, respectively), rat anti-K8 (Developmental Studies Hybridoma Bank, TROMA-I, 1:200 and 1:50 of supernatant for 2D and 3D studies, respectively), rabbit anti-E-cadherin (Cell Signaling, 3195, lot number 10, 1:400), goat anti-rabbit AlexaFluor (AF) 647 (Thermo Fisher Scientific, A21245, lot number 1805235, 1:500) and anti-rabbit horseradish peroxidase (HRP) (DAKO, P0448, lot number 20023997, 1:500). See supplementary Materials and Methods for further information and Lloyd-Lewis et al. (2016) for optimisation and validation studies.

### Animal models

All animal experimentation was carried out in accordance with the *Animal (Scientific Procedures) Act 1986*, the *European Union Directive 86/609*, and with local ethics committee approval. Mouse (*Mus musculus*) strains *R26^[CA]30^* (Kozar et al., 2013) (a kind gift from Prof. D. Winton, Cancer Research UK Cambridge Institute), *R26^Confetti^* (Livet et al., 2007) and *R26^CreERT2^* (Ventura et al., 2007) have previously been described. *R26^[CA]30^* experimental mice were hemi- or homozygous for *R26^[CA]30SYNbglA^*. Mice were analysed for β-glucosidase expression during adulthood (7-22-weeks). Multi-colour lineage-tracing studies were performed on mice that were hemizygous for both *R26^Confetti^* and *R26^CreERT2^* (*R26^Confetti^;R26^CreERT2^* mice). See supplementary Materials and Methods for further information.

### Induction of lineage tracing in *R26^Confetti^;R26^CreERT2^* mice

Tamoxifen was prepared in sunflower oil containing 10% ethanol. For lineage tracing during puberty in *R26^Confetti^;R26^CreERT2^* mice, labelling was induced at the onset of puberty (4 weeks of age) by a single intraperitoneal injection of tamoxifen (0.5 mg per mouse, ∼35 μg/g) and tissue was harvested from 7-week-old mice. Using this dose, mammary gland development appeared to progress unabated, as previously reported (Rios et al., 2014). For lineage-tracing in lactating *R26^Confetti^;R26^CreERT2^* mice, labelling was induced after puberty (12-14 weeks old) by a single intraperitoneal injection of tamoxifen (1 mg per mouse, ∼40-50 μg/g), which did not grossly affect alveolar development, as previously reported (Rios et al., 2014). After 10 days, female mice were mated with C57BL/6J male studs and lactating tissue was harvested between lactation days 4 and 5. For embryonic labelling, homozygous *R26^Confetti^* mice were mated with homozygous *R26^CreERT2^* mice. A single dose of tamoxifen (33 μg per g maternal body weight) containing progesterone (13 µg per g maternal body weight) was administered to pregnant mice via oral gavage at E16.5-17.5 (Li et al., 2016; Park et al., 2008). Using this dose, terminal end buds (TEBs) appeared morphologically normal and branching morphogenesis appeared normal via stereomicroscopy (Fig. S6). Mice were allowed to litter and tissue was collected from *R26^Confetti^;R26^CreERT2^* offspring 6 weeks after birth.

### Optical tissue clearing and whole-mount immunostaining

Fixed mammary tissue was cut into large pieces (∼15×15×2 mm) for immunostaining and tissue clearing, without any mechanical or enzymatic manipulation or microdissection. Optical tissue clearing was performed using either SeeDB (Ke et al., 2013) or a modified CUBIC (Reagent 1A) protocol (Susaki and Ueda, 2016), as previously described in detail (Lloyd-Lewis et al., 2016). Whole-mount immunostaining was performed prior to tissue clearing (SeeDB) or following immersion in CUBIC Reagent 1A, as previously described. See supplementary Materials and Methods for further information.

### Confocal microscopy

Optically clear tissues were imaged in their respective refractive index matching solutions in 35 mm glass-bottom MatTek dishes. Images were acquired using a Leica TCS SP8 inverted confocal microscope with 10×/0.4 or 20×/0.75 HC PL APO objective lenses. All colours (GFP, YFP, RFP and far red) were imaged for consistency and quantification. CFP-expressing clones were under-represented and were not routinely imaged (see Fig. S2). See supplementary Materials and Methods for further information.

### Whole-mount histochemistry

Detection of modified β-glucosidase expression in the mammary gland was performed as previously described (Davis et al., 2016). Briefly, excised mammary glands were fixed at room temperature for 4 h in NBF (10%). Tissue was heated to 65°C for 15 min in phosphate-buffered saline for endogenous β-glucosidase inactivation. Whole-mount mammary glands were incubated for 24 h at 50°C in a solution containing one part solution A [5-bromo-6-chloro-3-indolyl-β-D-glucopyranoside (1%) in dimethyl sulfoxide] and 25 parts solution B [magnesium chloride (0.02% w/v), potassium ferricyanide (0.096% w/v) and potassium ferrocyanide (0.13% w/v) in PBS]. After 24 h, the substrate was replenished and tissue incubated for an additional 24 h. Mammary glands were post-fixed in 10% NBF overnight at 4°C. Tissue clearing was performed using the CUBIC clearing protocol (Susaki et al., 2014), with methyl green counterstaining, as previously described (Davis et al., 2016; Lloyd-Lewis et al., 2016).

### Histology

For histological analysis of tissue from *R26^[CA]30SYNbglA^* mice, CUBIC-based tissue clearing was reversed by overnight incubation in PBS at 4°C. Paraffin processing was performed using a butanol clearing protocol, to maintain the histochemical magenta staining. Briefly, tissue was placed in a cassette and immersed in 70% ethanol (2 h), 96% ethanol (2 h), 100% ethanol (2 h) and finally transferred to n-butanol for 2 h before paraffin wax embedding. Paraffin wax-embedded sections (4-6 µm) were de-waxed in xylene (3×2 min washes) and processed as described above. Primary antibodies used for immunohistochemistry on paraffin slides were: rabbit anti-SMA (Abcam, ab5694, 1:200) and rabbit anti-E-cadherin (Cell Signaling, 3195, 1:50). Goat anti-rabbit HRP-conjugated secondary antibody (Jackson ImmunoResearch) was used at a dilution of 1:250.

### Clonal analysis method

A cell neighbour analysis was used to analyse labelling outcomes in this study and is described in detail in the supplementary Materials and Methods. Briefly, we created *z*-projections of randomly selected 3D image stacks containing label-positive cells. For all cells within each region, the lineage of the closest same-colour neighbour was recorded as either ‘same’ or ‘different’ by manual scoring. GFP, YFP, RFP and far red channels were imaged for each image sequence.

## Supplementary Material

Supplementary information
