# Supplementary material for: Neutral lineage tracing of proliferative embryonic and adult mammary stem/progenitor cells
Source: Development. 2018 Jul 25;145(14):dev164079. doi: 10.1242/dev.164079 (PMC6078330; doi:10.1242/dev.164079)
Supplement: Supplementary information [file develop-145-164079-s1.pdf]

SUPPLEMENTARY MATERIALS AND METHODS

Reagents

Triton X-100 was purchased from VWR International. Sucrose was purchased from Fisher Scientific. Imaging dishes were purchased from Ibidi. The following reagents were purchased from Sigma Aldrich: α-thioglycerol, 5-bromo-6-chloro-3-indolyl β-D-glucopyranoside, 3,3'-diaminobenzidine tetrahydrochloride, DMSO, fructose, neutral buffered formalin, 2,2',2"-nitrilotriethanol, N,N,N',N'-tetrakis(2-hydroxypropyl)ethylenediamine, potassium ferricyanide, potassium ferrocyanide, tamoxifen and urea.

Animals

All mice were sacrificed by dislocation of the neck or by terminal anesthesia. Eight mammary glands (pairs 2-5) were dissected from each mouse. Mammary tissue was fixed in 10% neutral buffered formalin (NBF) for 9 h at room temperature (Lloyd-Lewis et al., 2016). All animals were housed in individually ventilated cages under a 12:12 h light-dark cycle, with water and food available *ad libitum*. No mice were excluded from these studies. For studies using the $R26^{CreERT2}$;$R26^{Confetti}$ model, 3-5 mice were used based on the density of labelling at each developmental stage. Regions for analysis were randomly-selected from 3D image sequences.

Wholemount immunohistochemistry

Primary antibodies were diluted in blocking buffer at 4°C for 4 days with gentle agitation. Tissue was washed and incubated with secondary antibody conjugated to Alexa-Fluor 647 for 2 days before further washing in PBS and incubation with DAPI (10 µM) for 2-3 h. DAPI staining was needed to help identify and visualise ductal structures by confocal microscopy. This fluorescent stain spectrally overlaps with cyan fluorescent protein (CFP), however, CFP-expressing clones were underrepresented in $R26^{Confetti}$;$R26^{CreERT2}$ mice, as previously reported (Davis et al., 2016) and were thus excluded from further analyses.

Confocal microscopy

For standard 4-colour (GFP, YFP, RFP, AF647) imaging, laser power and gain were adjusted manually to give optimal fluorescence for each fluorophore with minimal photobleaching. Imaging depths were recorded from the top of the epithelial structure being imaged (typically 350 µm through the native fat pad). Image reconstructions were generated in ImageJ using the Bio-Formats plugin (National Institutes of Health) (Linkert et al., 2010; Schindelin et al., 2012). Denoising of 3D image stacks was performed in MATLAB (R2014a, The Mathoworks Inc.)(Boulanger et al., 2010).

Method of clonal analysis

Ductal elongation and side branching during puberty occur due to stem/progenitor cell proliferation within terminal end buds. As terminal end buds elongate, the progeny of stem/progenitor cell divisions are dropped along the length of the developing ducts, with mixing of clonal progeny. Stem/progenitor cell clones arising from labelling in the embryonic and pubertal epithelium are, thus, not contiguous. There exists conflicting reports as to whether ductal epithelial cells undergo significant proliferation during pubertal development (Scheele et al., 2017; Wang et al., 2015). EdU positive cells have been observed in ductal structures during puberty (Wang et al., 2015), and thus we chose a method of clone analysis that did not exclude the possibility of ductal proliferation and took into account the bona fide 3D nature of the mammary epithelium. Quantification was based off previous pair/patch-based analysis (Wuidart et al., 2016), with manual scoring (Rios et al., 2014; van Amerongen et al., 2012; Van Keymeulen et al., 2011). 3D image stacks of label positive regions were randomly selected based on image quality (and thus ability to accurately record cell lineage via cell morphology, topology and SMA positivity). For all cells within a region, the lineage of its closest same-colour neighbour was recorded in one of two bins: “same” or “different”. For example, if a luminal YFP+ cell was observed and its closest YFP+ neighbour (in x-y-z) was also luminal, this cell would be counted as “same” (potentially supporting unipotency of the clone). If, on the other hand, its closest YFP+ neighbour was basal, this would be counted as “different” (potentially supporting bipotency of the clone). Hundreds of cells were counted over different clones in different mice to obtain a cell neighbour lineage analysis.

This method of analysis makes the following assumption (based on the relatively low level of labelling and the multi-colour reporter system)—two proximal cells of the same colour within a specific region were derived from a common labelled ancestor. Thus, two luminal YFP+ cells counted as “same” may be distinct labelling events (thus this model may over-represent the percentage of possible unipotent clones). A luminal and basal YFP+ cell counted as “different” may also be from distinct labelling events (thus this model may also over-represent the proportion of possible bipotent clones, which was extremely small in this study).

Development • Supplementary information

SUPPLEMENTARY FIGURES

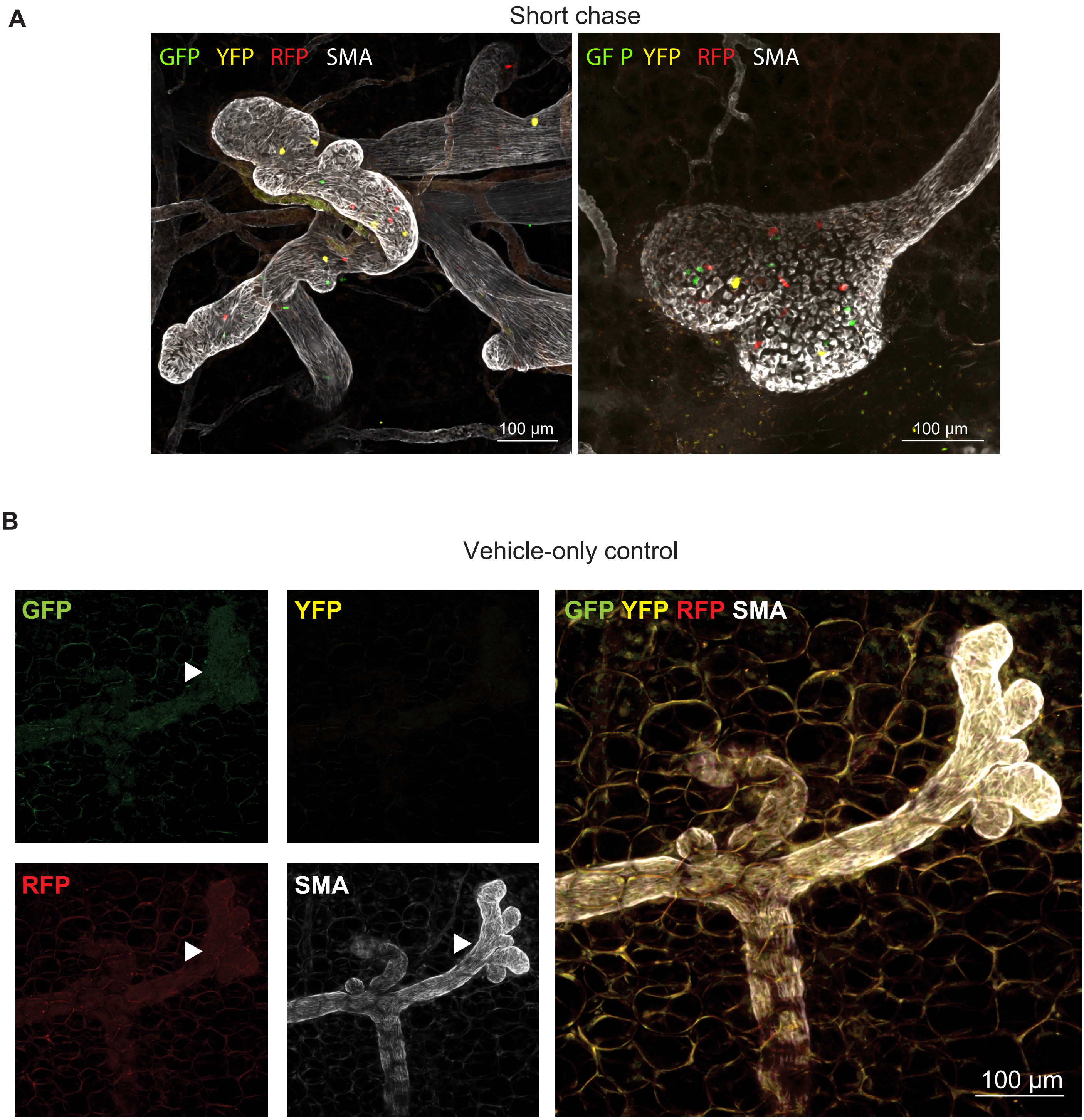

Fig. S1: Short chase and vehicle-only control for *R26*^{CreERT2};*R26*^{Confetti} pubertal mice. Images showing the level of FP expression following a short (2-day) chase (**A**) and the absence of labelling in mice injected with oil (vehicle) (**B**). Images show the maximum-intensity *z*-projection. Arrowheads show non-specific background fluorescence. Related to Fig. 1.

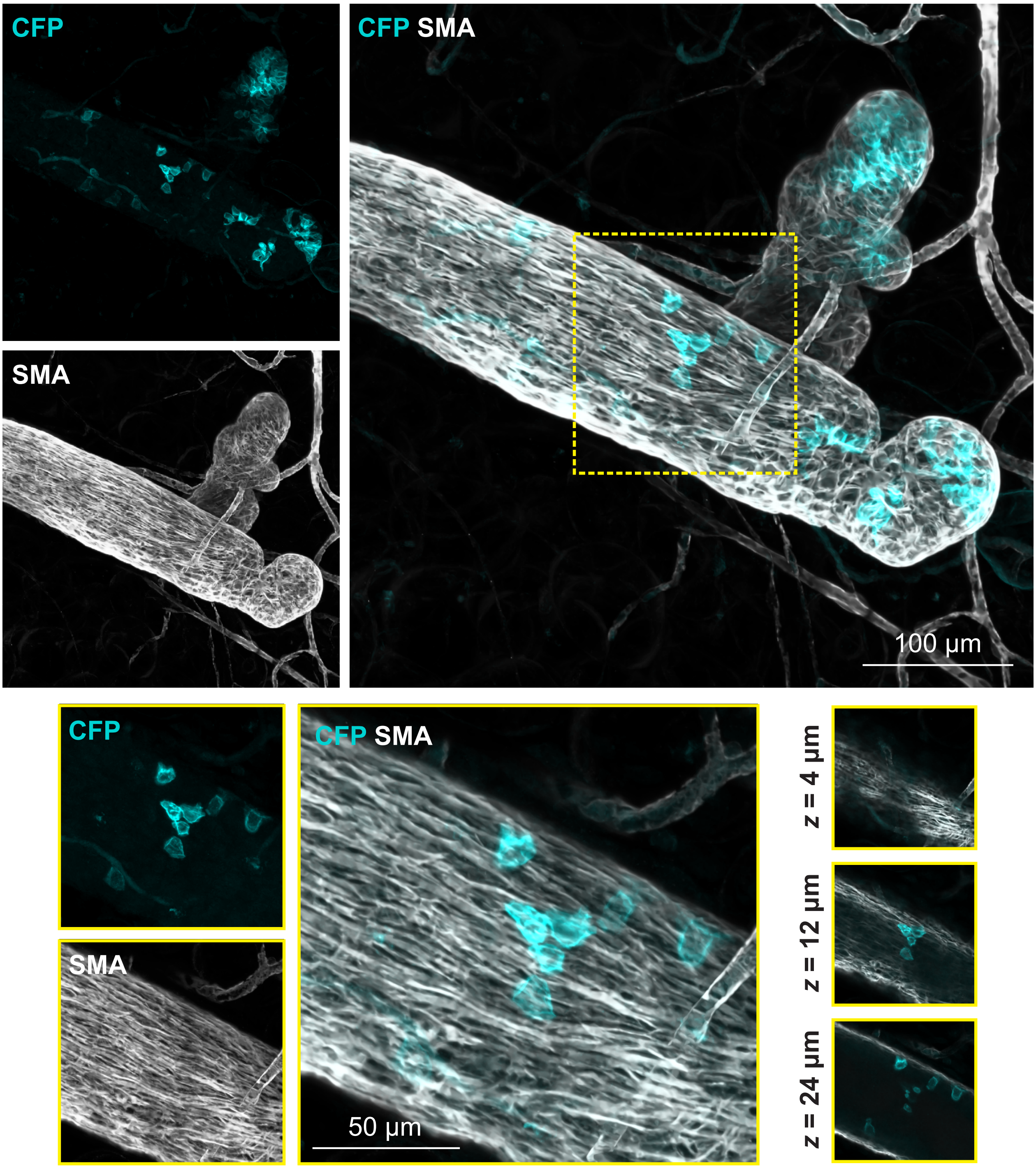

Fig. S2: Example a region containing rare CFP+ cells. Images show the maximum-intensity *z*-projection (single colour and overlay) and three optical slices of an ROI, demarcated inset (yellow box). Related to Fig. 1.

Development • Supplementary information

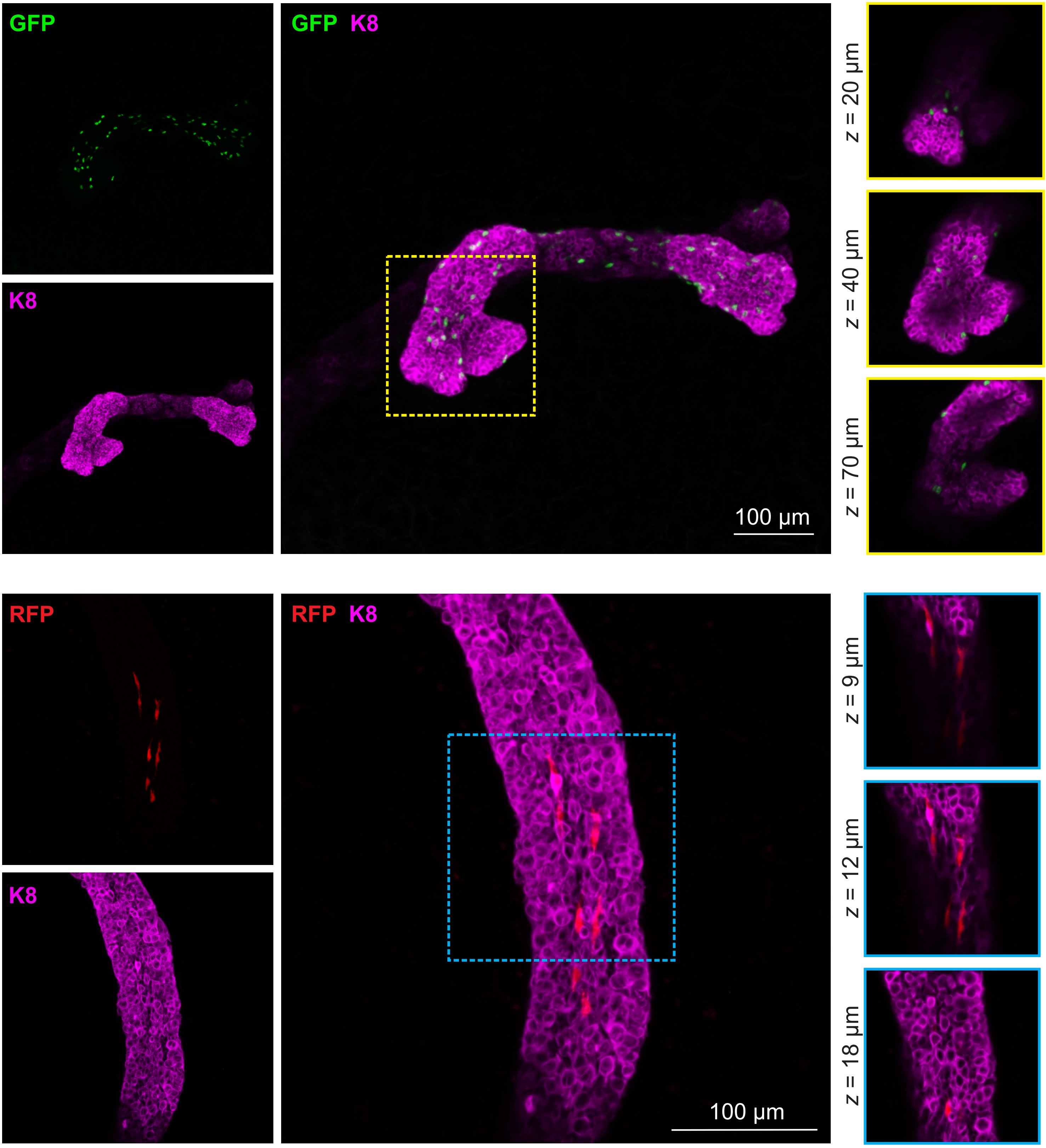

Fig. S3: Example of GFP+ (top) and RFP+ (bottom) single-colour branches. Images show the maximum-intensity *z*-projection (single colour and overlay) and three optical slices of a region-of-interest (ROI), demarcated inset. Related to Fig. 1.

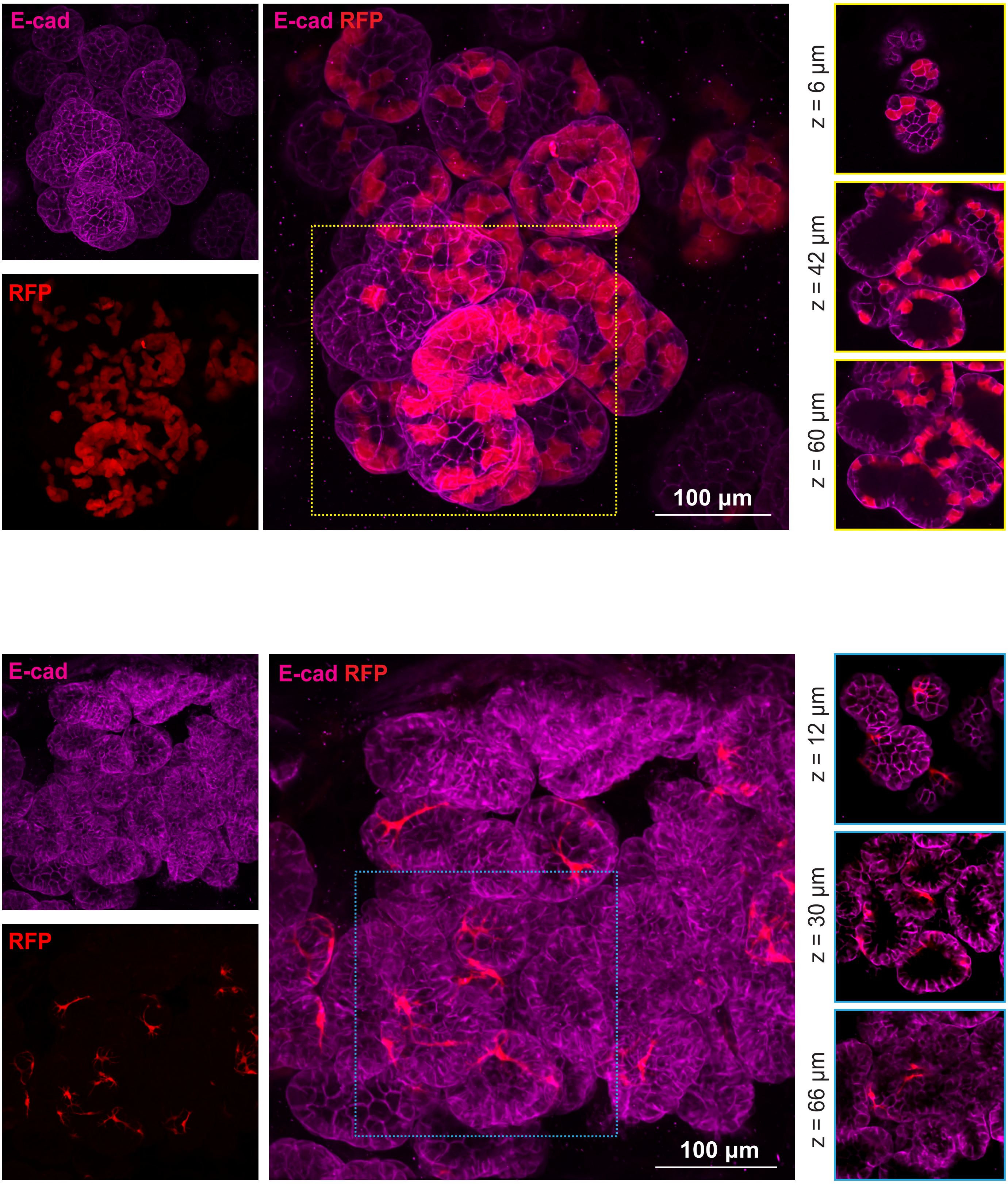

Fig. S4: Example of single-colour luminal (top) and basal (bottom) alveoli. Images show the maximum-intensity *z*-projection (single colour and overlay) and three optical slices of an ROI, demarcated inset. Related to Fig. 2.

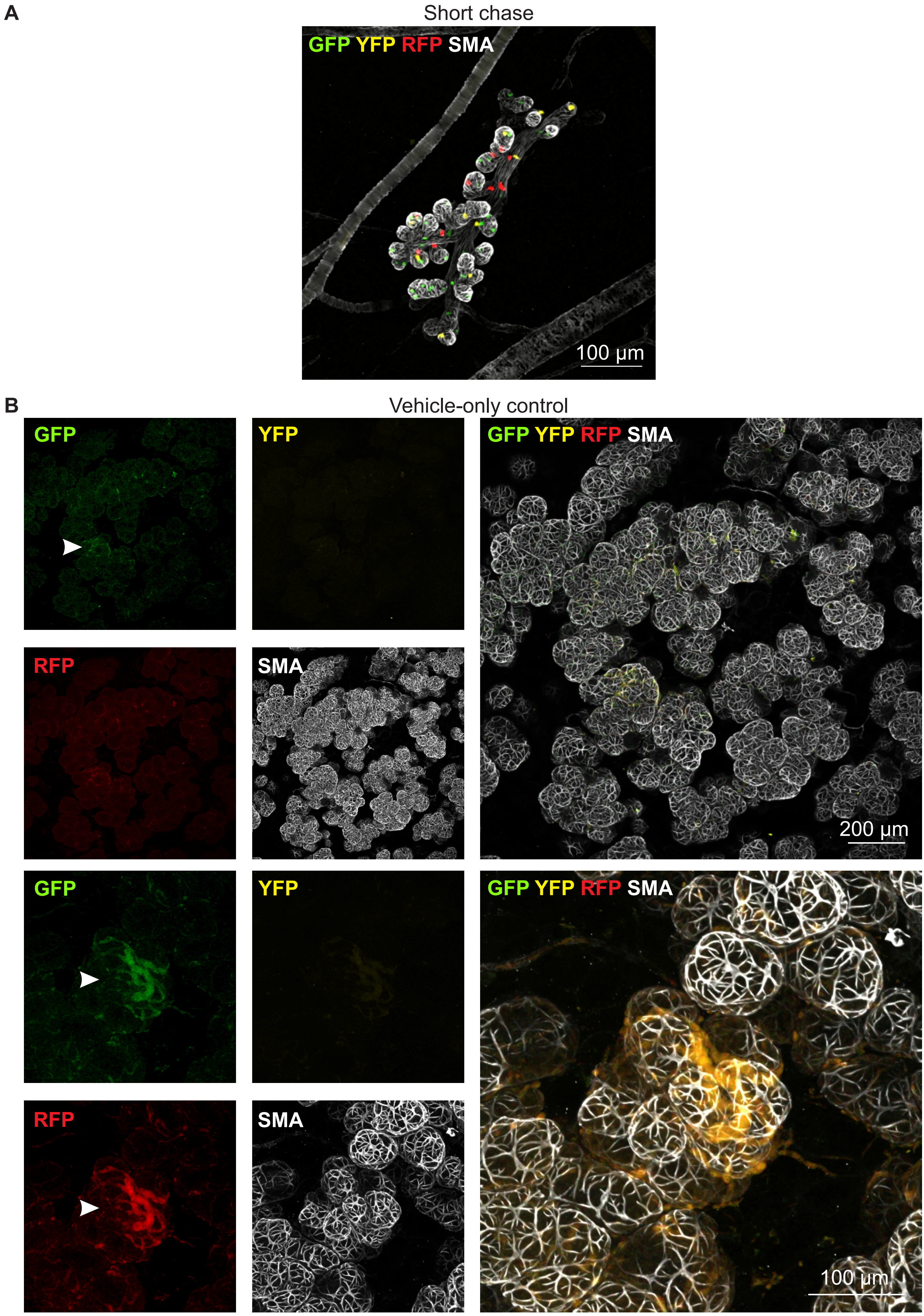
A
Short chase
GFP YFP RFP SMA
100 µm
B
Vehicle-only control
GFP
YFP
GFP YFP RFP SMA
RFP
SMA
200 µm
GFP
YFP
GFP YFP RFP SMA
RFP
SMA
100 µm

Fig. S5: Short chase control and vehicle-only control for *R26*^{CreERT2}*;R26*^{Confetti} lactating mice. Images showing the level of FP expression following a short (2-day) chase (**A**) and the absence of labelling in mice injected with oil (vehicle) (**B**). Images show the maximum-intensity *z*-projection. Arrowheads show non-specific background fluorescence. Related to Fig. 2.

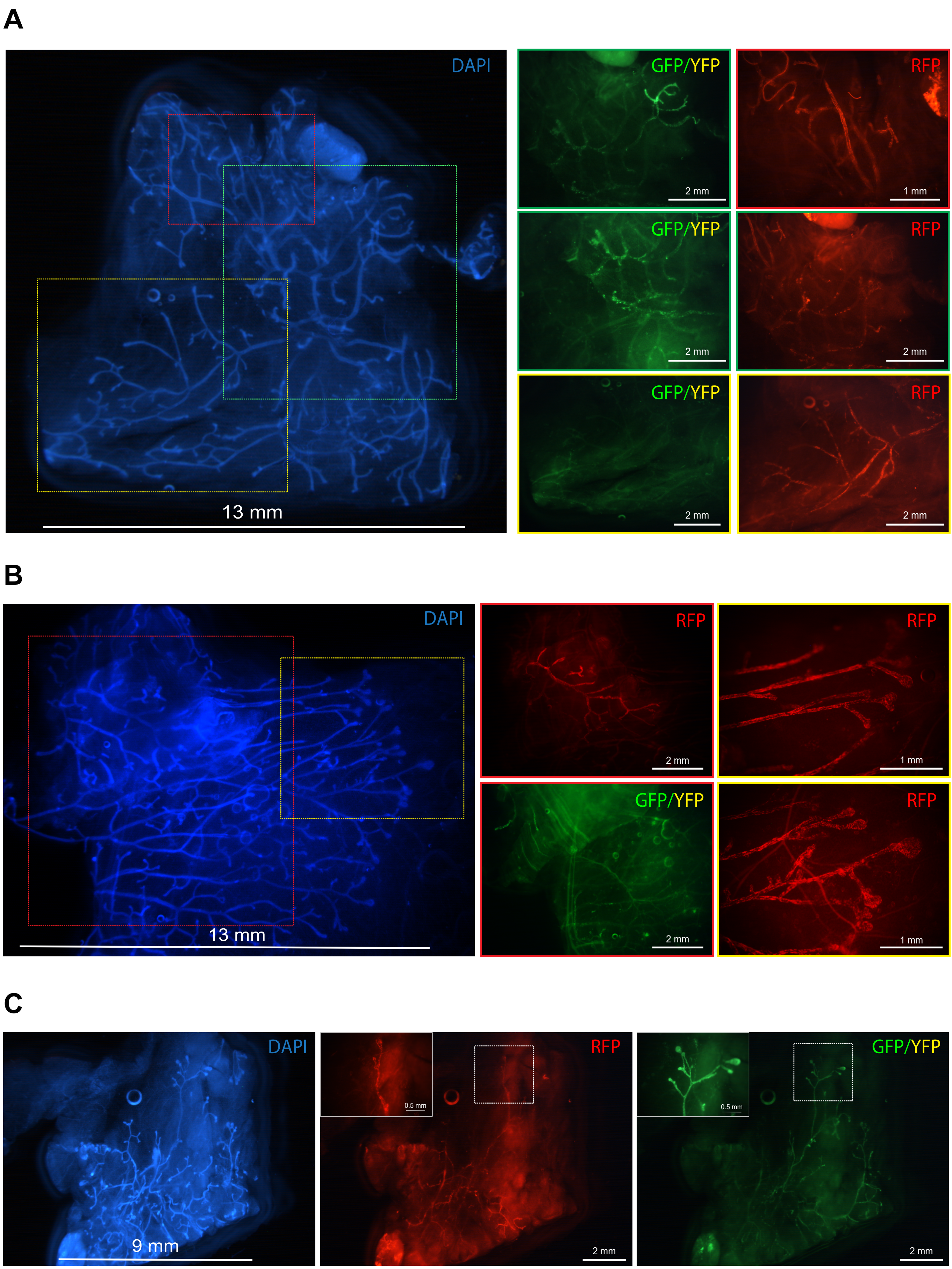

Fig. S6: Wholemount fluorescence (stereo microscope) images of the mammary ductal network revealing the extent of labelling observed in 6-week old mice labelled in utero. Related to Fig. 3.

Development • Supplementary information

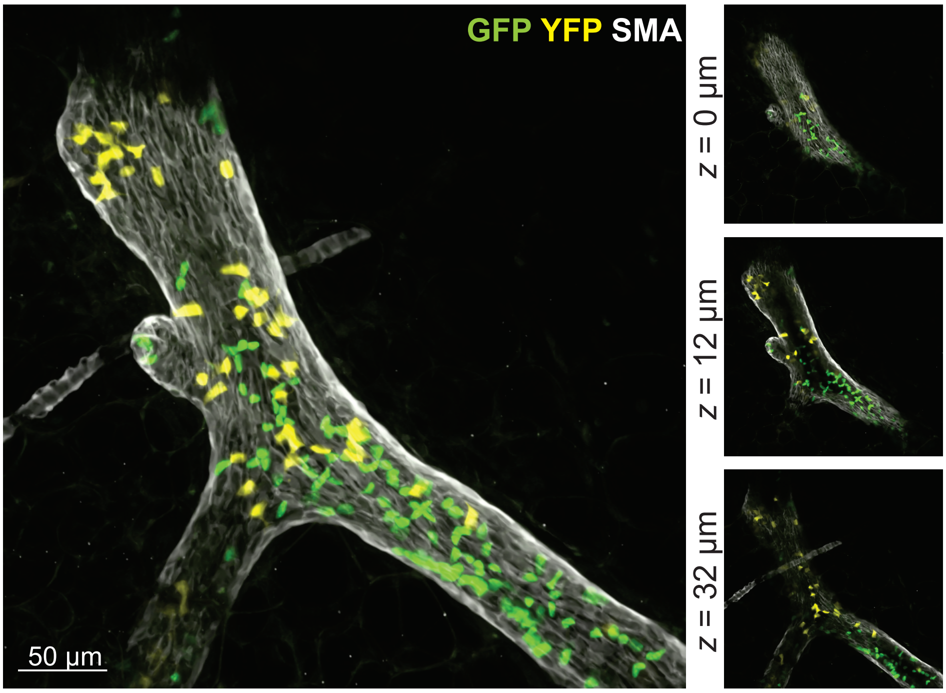

Fig. S7: Example of multi-colour distal branches in mammary glands of 6-week old mice labelled in utero. Related to Fig. 3.

Development • Supplementary information
